# Trichobezoar: Ravenous for Hair

**DOI:** 10.5005/jp-journals-10018-1271

**Published:** 2018-05-01

**Authors:** Akshay Prasad, Atul Jain, Akash Gupta, Aman Kamra

**Affiliations:** 1 Department of Surgery, Subharti Medical College, Meerut, Uttar Pradesh, India; 2 Department of Surgery, ESI Post Graduate Institute of Medical Science and Research & Hospital, Basaidarapur, New Delhi, India

**Keywords:** Bezoar, Rapunzel syndrome, Trichobezoars, Trichophagia.

## Abstract

Trichophagia is the habit of chewing hair. On an average, only 1% patients with trichophagia develop trichobezoar. They are usually symptomless until they reach a large size. It is common in young females usually with an underlying psychiatric disorder. The gold standard for diagnosis is upper gastrointestinal endoscopy (UGIE). Here we present a case of trichobezoar in an 11-year-old girl who presented in the emergency with complaint of pain abdomen and symptoms of obstruction.

**Key messages:**

A high index of suspicion among physician can lead to early diagnosis and management in these cases (trichobezoar).

**How to cite this article:** Prasad A, Jain A, Gupta A, Kamra A. Trichobezoar: Ravenous for Hair. Euroasian J Hepato-Gastroenterol 2018;8(1):97-98.

## INTRODUCTION

Bezoar is a collection of foreign material in the intestinal tract (usually stomach). This term is believed to originate from the Arabic word “Badzehr,” Persian word “Padzahr,” or Turkish word “Panzehir,” all of which mean substance that act as “antidote” or counterpoison.^1^ It is common in young females usually with mental retardation and an underlying psychiatric disorder, who chew and swallow their hair, vegetable fibers, etc.

Bezoars can be classified according to the content, such as trichobezoar (hair), phytobezoar (vegetable fibers), lactobezoar (milk products), pharmacobezoar (drugs), diospyrobezoars (persimmon fibers), cotton bezoars (cotton fibers).^2^

Trichobezoars were first described by Baudomont in 1779.^3^ Trichopha gia is the habit of chewing the hair. On an average, only 1% patients with trichophagia develop trichobezoar.^4-6^ The slippery surface of the hair tufts resists the gastric peristaltic propulsion and tends to collect in the gastric mucosal folds. As more and more hair accumulates, a ball of hair is formed, which becomes too large to escape and results in gastric atony and subsequently takes the shape of stomach.^7-9^

Here we present a case of trichobezoar in an 11-year-old girl who had the habit of chewing hairs and who presented in the emergency with pain in abdomen and symptoms of obstruction.

## CASE REPORT

An 11-year-old thin-built, anemic girl was brought to the emergency with complaint of pain in upper abdomen and recurrent vomiting since 1 week. Abdominal examination revealed a nontender lump in the epigastric region, which was moving with respiration. Rest examination was normal. In laboratory examination, microcytic hypochromic anemia was seen; other parameters were normal. Parents gave history of disturbed behavior of child for past few months and decreased appetite. Sibling of the patient had seen her chewing her hairs infrequently, but did not think of it to be a major problem.

**Fig. 1:**
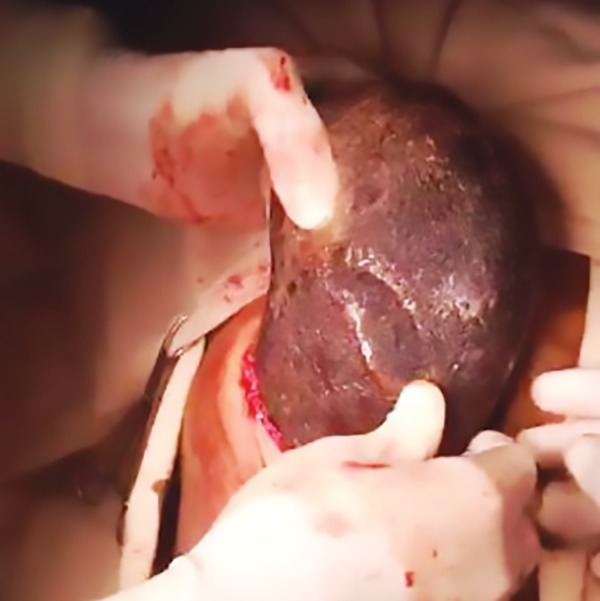
Hair ball mass delivered through gastrotomy wound

**Fig. 2:**
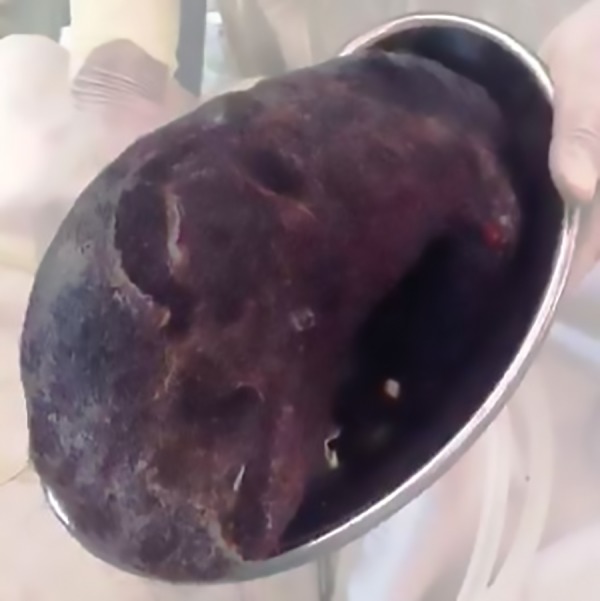
Large trichobezoar

She was initially admitted in other hospitals for this, where conservative management was given, but there was no relief. Along with other routine investigations, UGIE was done in which there was a large blackish mass in the stomach and the sample taken from it showed mostly hairs. Based on the history and examination, diagnosis of trichobezoar was made and patient was taken for surgery. Anterior gastrotomy was done (Fig. 1) and large hair ball mass (Fig. 2) which was occupying the whole stomach and extending into duodenum was removed. Postoperative period was uneventful and she was discharged after psychiatric consultation. After 6 months follow-up, patient is doing well.

## DISCUSSION

Rapunzel syndrome was first described in the literature by Vaughan et al in 1968^9^ in which a dense compact mass of hair (trichobezoar) was found in the stomach with extension into intestine through duodenum in patients with a history of psychiatric disorder. The name “Rapunzel” syndrome comes from the Grimm Brothers’ fairy tale of a 12-year-old princess who was shut into a tower with neither stairs nor doors by an enchantress who climbed up the tower’s walls with the help of Rapunzel’s long tresses.^10^

Trichobezoars are usually symptomless until they reach a large size. Clinical presentation is deceptive and vague, ranging from an abdominal mass to gastrointestinal symptoms, wasting and cachexia. Large bezoars are often palpable and may be indentable (Lamerton’s sign).

Severe halitosis and patchy alopecia provide clues on physical examination. Imaging may show the bezoar as a mass or filling defect. The gold standard for diagnosis is UGIE. In addition to direct visualization, one can take sample and therapeutic intervention can also be done in some cases.

Complications of trichobezoars include gastrointestinal obstruction (26%), bleeding (10%), perforation, malabsorption, and nutritional deficiencies. If bezoars are left without treatment, the mortality rate can reach 30% because of the associated complications.^1^

In our case, the patient had psychiatric symptoms but the family did not take it seriously. The clinical presentation was also vague, which made the diagnosis of trichobezoar difficult in the initial stage. The finding of UGIE led to the diagnosis and then surgery was planned as endoscopic removal was not possible.

## CONCLUSION

There should be awareness among the general public for any mental disturbance or altered behavior, which should be consulted with the physician or psychiatrist and treatment should be started before any delay leads to complications, whether physical, mental, or social.

A high index of suspicion among physicians can lead to early diagnosis and management in these cases (trichobezoar).
